# Highly Conductive PEO/PAN-Based SN-Containing Electrospun Membranes as Solid Polymer Electrolytes

**DOI:** 10.3390/membranes15070196

**Published:** 2025-06-30

**Authors:** Anna Maria Kirchberger, Patrick Walke, Janio Venturini, Leo van Wüllen, Tom Nilges

**Affiliations:** 1 TUM School of Natural Sciences, Technische Universität München, Lichtenbergstr. 4, 85748 Garching bei München, Germany; anna.koehlmeier@tum.de (A.M.K.); janiovj@gmail.com (J.V.); 2 TUMint.Energy Research GmbH, Lichtenbergstr. 4, 85748 Garching bei München, Germany; 3 Institute of Physics, University of Augsburg, Universitätsstraße 1, 86159 Augsburg, Germany

**Keywords:** solid polymer electrolytes, all-solid-state batteries, electrospinning, conductivity

## Abstract

Solid polymer electrolytes (SPEs) have garnered significant attention due to their potential in all-solid-state batteries (ASSBs). However, adoption remains constrained by challenges such as low thermal stability and limited ionic conductivity. Here, we report on an electrospun (PAN/PEO)- conductive salt (LiBF_4_) system, where the influence of varying polyacrylonitrile (PAN) and polyethylene oxide (PEO) ratios, along with different plasticizer concentrations, is evaluated. Notably, the 50:50 PAN/PEO sample exhibited the highest ionic conductivity, reaching 1∙10^−2^ S/cm at 55 °C. This system also balanced conductivity and processability. Succinonitrile (SN) significantly influenced the morphology and conductivity. Samples with increased SN content showed enhanced capacity in symmetrical cells, achieving ~140 mAs/cm^2^ for an 18:9:1 polymer (PAN/PEO):SN:conductive salt (LiBF_4_) composition. The enhanced lithium-ion conductivity of the electrospun blend is attributed to the deliberate use of an unmixable PAN–PEO system. Their immiscibility creates well-defined interfacial regions within fibers, acting as efficient lithium-ion pathways. These findings support electrospun polymer blends as promising candidates for high-performance SPEs for ASSB development.

## 1. Introduction

With the ongoing strides in energy science and technology, new options for energy storage have gained increasing importance. In order to reduce the number of combustion engine vehicles and thereby decrease CO_2_ emissions, the mobility sector garnered great attention in the last years, particularly in the case of battery-powered electric vehicles. Lithium-ion batteries (LIBs) are the most-used medium for energy storage; however, liquid-electrolyte LIBs still lack stability and often have safety concerns during application. Major problems still exist in these devices, mostly regarding their cycle stability—limiting the lifetime and performance of the system—and, most importantly, the safety requirements [1,2,3]. The high reactivity of the anode towards the electrolyte can cause the formation of dendrites, which may pierce the separator and cause short circuits and thermal runaways due to the flammability of the solvents [3]. Decomposition products may also occur in liquid-based LIBs and generate the typical swelling of defective batteries [1,3].

Alternative electrolytes are intensely examined to minimize this potential hazard. The substitution of liquid by solid polymer electrolytes (SPEs) may lead to significant improvements regarding the safety of these electrical devices. The high mechanical flexibility of SPEs enables their use in multiple types of battery cell formats [4]. This flexibility allows the option for smaller, lighter, and safer cells which offer higher energy densities, better cycle stability, and the potential for flexible geometries. In order to realize an SPE-based all-solid-state battery (ASSB), the electrolyte must provide high ionic conductivity, good mechanical processability, and support high cycle stability [5,6].

In the field of solid-state electrolytes, several systems have been investigated in the past. Firstly, there are non-polymer based inorganic solid electrolytes like perovskite-type [7], Na super ionic conductor (NASICON) [8,9,10,11], garnet-type [12,13], Li super ionic conductors (LISICON) [14,15], lithium phosphorus oxynitride (LIPON), [16] and sulfide-type materials [17], just to name a few.

A large field of interest deals with polymer electrolytes, which offer the discussed advantages of flexibility, easy processability, and reduced weight [18,19,20,21]. Polymer electrolytes like PEO have been intensively studied since the late 1970s after the first report by Farrington et al. [22] PEO is one of the most prominent and well-examined conductive polymers if conductive salts are added. The conduction takes place via the separation of the Li^+^[A]^−^ contact ion pair complex through the coordination of Li^+^ by the O-functionalities of PEO. Ion hopping along and between the chains is facilitated by the coordination via the ether groups and enhanced by the chain motion of the PEO backbone. Nevertheless, the resulting conductivity is still too low in such polymer-conductive salt systems [23]. As a result, much effort is directed toward possible additives, e.g., fillers or plasticizers, that could solve this conductivity issue. Besides the polymer optimization itself, there are various studies on the use of fillers such as TiO_2_, Al_2_O_3_, SiO_2_, or Li_0.33_La_0.557_TiO_3_ to enhance the conductivity of polymer electrolytes via surface percolation effects [24,25,26]. Another promising method to enhance key properties of polymers is the addition of plasticizers, for instance, succinonitrile (SN), which is known to have a positive impact on physical properties like crystallinity, conductivity, and chain mobility in PEO [27,28,29].

PAN (polyacrylonitrile) is an alternative building block for SPEs, which may not contribute to the overall conductivity but offers matrix-like properties and enhanced thermal stability due to its high melting point [30]. As a non-conductive additive, PAN has shown benefits for the overall system performance due to its matrix character and filling properties [31,32]. The coordination of the lithium ions in PAN is reduced in comparison to PEO-like polymers [33]. In the past, PEO/PAN-polymer blends were investigated but the solvent content was so high that they were classified more as a gel electrolyte. The utilization of gel electrolytes in general is limited by dimensional and mechanical stability issues, where size, shape, or structure under varying conditions (e.g., temperature fluctuations, humidity, or mechanical stresses) change, and furthermore possibly lead to the passivation of the lithium electrode upon contact [34].

Electrospinning as a processing method for the fabrication of fiber membranes, offers several advantages for the development of polymer electrolytes due to its ability to create nanofibrous structures that enhance material performance. This process results in a high surface area to volume ratio, which can improve ionic conductivity, while the mechanical properties are defined by the interwinding fiber arrangement, compared to traditional bulk materials. Moreover, the controlled fabrication of electrospun membranes allows for the fine-tuning of polymer morphology and structure. While solution casting typically leads to phase separation during the drying process for polymers with different polarities, like in the case of PEO and PAN, the electrospun approach facilitates better polymer mixing. This aspect tends to improve the overall functionality and performance of the material.

In this work, blend polymer electrolytes were developed with the goal of finding a highly conductive composite solid polymer electrolyte featuring reasonable electrochemical stability and performance. We selected the combination PAN/PEO, LiBF_4_ as a relatively moisture-insensitive conductive salt, and succinonitrile (SN) as an established PEO plasticizer additive in this SPE study. The polymer composition and plasticizer content were varied to identify the best-performing SPE system.

## 2. Materials and Methods

The listed compositions for the systems are starting material ratios and do not represent their final composition. The given SN content, therefore, represents the maximum plasticizer content for each system. Propylene carbonate (PC) was used as a co-solvent and phase compatibilizer for PAN processing and can also be present in the final product. Due to the ES processing and drying processes applied to all samples, we expect certain evaporation of the volatile starting materials (data concerning safety data sheets for SN: Melting point 50–60 °C, boiling point 265–267 °C, vapor pressure at 25 °C 0.5 hPa; data for PC: melting point −55 °C, boiling point 240 °C, vapor pressure at 25 °C 0.06 hPa). This evaporation is dependent on many parameters like evaporation time during ES, resulting fiber thickness after ES, drying effectiveness, and evaporation during post-ES processes [35]. We, therefore, decided to discuss each system based on its starting composition but being aware that the plasticizer and additive content should be lower than suggested by the starting composition. The solid polymer electrolytes were analyzed regarding electrochemical performance and crystallinity.

PC was introduced into the MeCN/DMSO processing mixture to fine-tune the blend’s mixability, enhancing the phase compatibility between PEO and PAN. Owing to its volatile nature, the propylene carbonate is effectively removed during drying [35]. This improved phase compatibility (before drying), when combined with the unique benefits of electrospinning, culminates in a solid polymer electrolyte with enhanced ionic conductivity and mechanical properties. To further improve the performance of the PEO/PAN blend, succinonitrile (SN) was incorporated as a plasticizer. Its role is to suppress the crystallization of PEO, thereby enhancing its amorphous character. This suppression of crystallinity facilitates increased chain mobility, promoting lithium-ion hopping and ultimately improving the ionic conductivity of the solid polymer electrolyte.

### 2.1. Electrospinning (ES)

The polymer solutions were prepared in the glovebox in a suitable glass vessel with a stirring magnet. Amounts of all starting materials are given in Table 1. In the first step polyacrylonitrile (PAN, Sigma Aldrich, Merck KGaA, Darmstadt, Deutschland, 150,000 g/mol), propylene carbonate (PC, Sigma Aldrich, battery grade, ≥99%, acid < 10 ppm, H_2_O < 10 ppm), and a certain amount of dimethyl sulfoxide (DMSO, Honeywell, Honeywell Chemicals, Germany, dry ≥ 99.9%, ≤0.02% H_2_O) were added to the flask, in amounts depending on the desired composition (Table 1). The reactants were stirred until a homogeneous solution was achieved, after which a specified amount of acetonitrile (MeCN, obtained from a solvent purification system (MB-SPS; MBraun INTERTGAS-SYSTEME GmbH, Garching, Germany) was added (Table 1). Subsequently, polyethylene oxide (PEO, Sigma Aldrich, Merck KGaA, Darmstadt, Deutschland, 300,000 g/mol) and succinonitrile (SN, Sigma Aldrich, Merck KGaA, Darmstadt, Deutschland, 99%) were added to the solution. The mixture was stirred overnight. The conductive salt LiBF_4_ (Sigma Aldrich, Merck KGaA, Darmstadt, Deutschland, ≥98%, acid < 200 ppm, anhydrous) was added 2 h before the electrospinning process was initiated. All steps were carried out under dry conditions in an inert atmosphere (O_2_ < 0.1 ppm, H_2_O < 0.1 ppm). Table 1 contains detailed information on the quantities of starting materials, solvents, and reactants.

During electrospinning, a voltage of 18 to 20 kV was applied. The distance between the tip of the cannula and the grounded collector averaged 20 cm, and the solution was pumped at a feed rate of 1.5–3 mL. A static ring collector was used to deposit the fibers. The membranes were dried in a desiccator on a Schlenk line for 16 h. Afterward, the membranes were transferred and stored in the Glovebox. The membranes were created through electrospinning using a custom-built electrospinning apparatus, as detailed in previous literature [36].

The crystallinity of all samples was assessed via powder X-ray diffraction using an STOE STADIP diffractometer (Stoe & Cie GmbH, Darmstadt, Germany) using Cu-K_α1_ radiation (*λ* = 1.54051 Å). The equipment was operated with a germanium monochromator and a DECTRIS Mythen 1K (Dectris AG, Baden-Daetwill, Switzerland) semiconductor detector system. The measurement was conducted between 5 and 80° (2*θ*). A disc with a diameter of 10 mm was punched out of the membrane and positioned with Scotch Magic Tape^®^ (Bürobedarf, Böttcher AG, Zöllnitz, Germany) in a flat-bed sample holder. All measurements were run at room temperature.

### 2.2. Thermal Analysis

The thermal analysis of the membranes and reactants was conducted using a differential scanning calorimeter (DSC, Netzsch Maia DSC 200 F3, Netzsch Gruppe, Selb, Germany) in an aluminum crucible in a temperature range of 123–523 K, with a heating rate of 10 K/min under continuous nitrogen flow.

### 2.3. Scanning Electron Microscopy (SEM)

For the fiber images, a scanning electron microscope (SEM) JSM-IT200 InTouchScope^TM^ from JEOL (JEOL (Germany) GmbH, Freising, Germany) was used. The samples were taped to a graphite sample holder inside a glovebox and brought into the vacuum chamber of the SEM. An acceleration voltage of 1–10 kV was applied.

### 2.4. Potentiostatic Electrochemical Impedance Spectroscopy (PEIS)

The electrochemical analysis was operated with a Metrohm Autolab B.V. PGSTAT204 potentiostat (Metrohm Deutschland GmbH & Co. KG, Filderstadt, Germany) including an FRA 32 *M* module and a cell setup by rhd with a TSC standard battery cell and stainless steel electrodes. The membrane was punched out in the shape of a disc with a 10 mm diameter and placed between the stainless steel electrodes; the measurement area *a* is 0.50265 cm^2^. PEIS data was collected using an amplitude of 20 mV, in the frequency range of 1 MHz to 0.1 Hz, at temperatures from 293 to 328 K, in steps of 5 K. The thickness *d* of the samples was determined after the measurements with a micrometer screw (Holex, 0–25 mm, 0.001 mm accuracy) and they varied between 70 µm and 110 µm. Conductivities were calculated using σ = (1/R)∙(d/a), where the resistance (R), the thickness (d), and the area (a) were taken into account. We assumed full contact with the electrodes, neglecting the tortuosity and lower contact area of fiber membranes to the electrodes. The resulting Nyquist plots were analyzed using the RelaxIS 3 software by rhd instruments.

### 2.5. Cyclic Voltammetry

Cyclic voltammetry was measured in standard 2032 coin cells with a VMP3 potentiostate by Biologic (BioLogic, Seyssinet-Pariset, France). The coin cells were closed by an HS-HCR2 coin cell press of Hohsen Corporation (Hohsen Corp. Osaka, Japan). All cells were prepared in the glovebox under an argon atmosphere (O_2_ < 0.1 ppm; H_2_O < 0.1 ppm). No additional additives or electrolytes were used.

The cells contain lithium metal electrodes on both sides, with a diameter of 14 mm, and membranes with a diameter of 17 mm to reduce the risk of short circuits before the cycling process. The cells were cycled between −1 and 1 V with a rate of 0.1 mV at r.t. against the reference.

### 2.6. Raman Spectroscopy

Raman spectra of the membranes were obtained by a SENTERRA Spectrometer (BRUKEROPTICS GmbH, Ettlingen, Germany) equipped with a 785 nm laser, 1% power (1 mW), and an integration time between 5 and 10 s.

## 3. Results and Discussion

**Figure 1:**
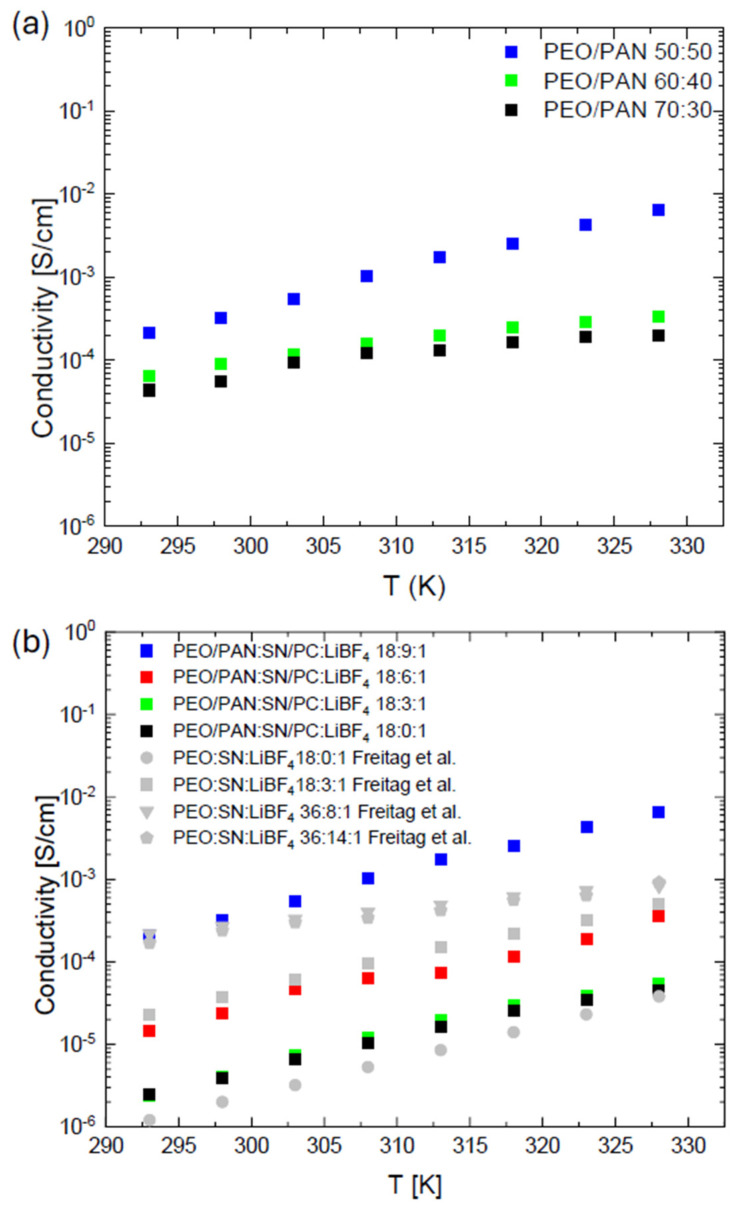
(**a**) Conductivity of membranes with different PEO/PAN ratios and a starting composition of (PEO/PAN):plasticizer: salt (18:9:1). The ratios of PEO to PAN in wt% were 50:50 (blue squares), 60:40 (green squares), and 70:30 (back squares). A trend towards lower conductivity for compositions with higher PEO amounts becomes obvious. (**b**) Conductivity data of various PEO/PAN:plasticizer:LiBF_4_ systems. Data reported by Freitag et al. for PEO:plasticizer:LiBF_4_ systems are denoted in grey [36]. The plasticizer-free PEO/PAN system (18:0:1, black squares) shows slightly better conductivity than the PEO system (grey dots). Upon plasticizer increases approximately the same conductivities are observed for the PEO/PAN system (18:6:1, red squares) and the PEO (18:3:1, grey squares) system. Reaching a plasticizer content of (18:9:1) in the PAN/PEO system, the conductivity overcomes the ones reported in Freitag et al. [36].

Blend polymer electrolytes composed of PEO and PAN were investigated in this work to produce a thermally and electrochemically stable, highly conductive, and tunable SPE. Electrospun PEO is a well-studied material for polymer electrolytes, given its ionic conduction, wide availability, and simple processability. Selected PEO systems are summarized in Figure 1b.

Nevertheless, there is wide room for improvement in its maximal conductivity, electrochemical stability, and thermal operation range, with the latter being rather narrow due to the low melting point of PEO, at around 55 °C. The addition of PAN could extend the thermal operation range by building a thermally stable scaffold for the polymer electrolyte, as pure PAN decomposes at 280 °C without previous melting.

In the following, we will report on physical, electrochemical, and morphological property determination and optimization using numerous methods and techniques. We focus on the electrochemical investigation of various polymer systems, varying the polymer content and composition, followed by a study of the influence of the plasticizer content on the previously identified, optimized polymer systems.

The PEO/PAN ratio was varied in a range of 70:30, 60:40, and 50:50 wt% in order to investigate the effect of PAN as a non-conductive and non-coordinating polymer to conductive PEO. Figure 1a depicts the electrical conductivity of the PEO/PAN solid polymer electrolytes produced with different polymer ratios. We decided to measure conductivity always up to 55 °C in order to stay in the solid PEO regime and to make it comparable to reported ES-PEO systems. Measurements at higher temperatures are currently underway and will be reported in an additional study.

We selected a polymer:conductive salt additive content of 18:1 in order to make it comparable to electrospun PEO:LiBF_4_ membranes reported by Freitag et al. in the literature [33].

The samples with high PEO fractions yield very similar results, with conductivities in the order of 10^−5^ S/cm and very little temperature dependence. The highest conductivity in this measurement is achieved by the 50:50 wt% PEO/PAN composition, with 1.0∙10^−4^ S/cm at room temperature and 1.0∙10^−2^ S/cm (or 10 mS/cm) at 55 °C. This composition with the highest fraction of PAN offers the best potential for further studies. Compositions with even higher amounts of PAN were also tested. Above a 50 wt% content of PAN, severe phase segregation occurred and proper fiber formation was not possible during the ES process. According to the reported synthesis procedure, the addition of PAN resulted in direct precipitation of the former dissolved PEO in that case. The r.t. conductivity of a pure electrospun PAN membrane containing PC as plasticizer (PAN:PC:LiBF_4_ 18:3:1) was below the detection limit of the used setup (<10^−9^ S/cm) for most of the tested compositions. A conductivity of 7.6·10^−8^ S/cm at 50 °C was determined for the composition 18:3:1 PAN:PC:LiBF_4_, approximately five orders of magnitude lower than that of the blended PEO/PAN membranes at the same temperature.

Let us now compare the PEO/PAN to pure PEO systems. The addition of 50 wt% PAN to PEO to an electrospun membrane for a comparable polymer-to-conductive salt ratio of 18:1 led to a slight increase in the conductivity (see Figure 1, bottom). At r.t., we determined a conductivity of 1.2·10^−6^ S/cm for the plasticizer-free PEO/PAN (18:0:1) system while the pure PEO (18:0:1) system reached 10^−6^ S/cm. This finding is surprising because we significantly reduced the conductive PEO phase content in the fibers. According to a previous study, PAN does not contribute to overall conductivity and only acts like a non-conductive matrix [33]. It seemed to be the case that the oriented PEO arrangement in the fibers may play a role. We varied the plasticizer content for the best performing PEO/PAN (50:50) system in order to evaluate the conductivity change upon plasticizer increase. Data for PEO/PAN (50:50) systems with (18:X:1) starting composition are denoted in Figure 1b). Upon plasticizer increase, we observe a continuous increase in the conductivity, which is a known phenomenon due to the increased chain mobility and reduced crystallinity (see later on). We found comparable conductivities for X = 6 plasticizer content that reaches the conductivities of a pure PEO (18:3:1) system. This slight discrepancy can either be due to a different plasticizing behavior in the PEO/PAN system, but a more likely explanation is the slight evaporation of SN during the electrospinning process which was also observed in a previous study [35]. Interestingly, despite the slightly different conditions and composition of the two systems, it seems to be the case that the PAN incorporation does not negatively influence overall ion conductivity. Increasing the plasticizer content further to X = 9, we could reach the same conductivities at r.t. as for the best performing PEO:SN:LiBF_4_ (36:8:1) system reported earlier on (see Figure 1b). At elevated temperatures, our PEO/PAN 50:50 (18:9:1) system outperforms the latter-mentioned PEO system. Overall, the incorporation of non-conducting PAN into PEO is beneficial for conductivity. A reason for this intriguing finding will be reported later on.

**Figure 2:**
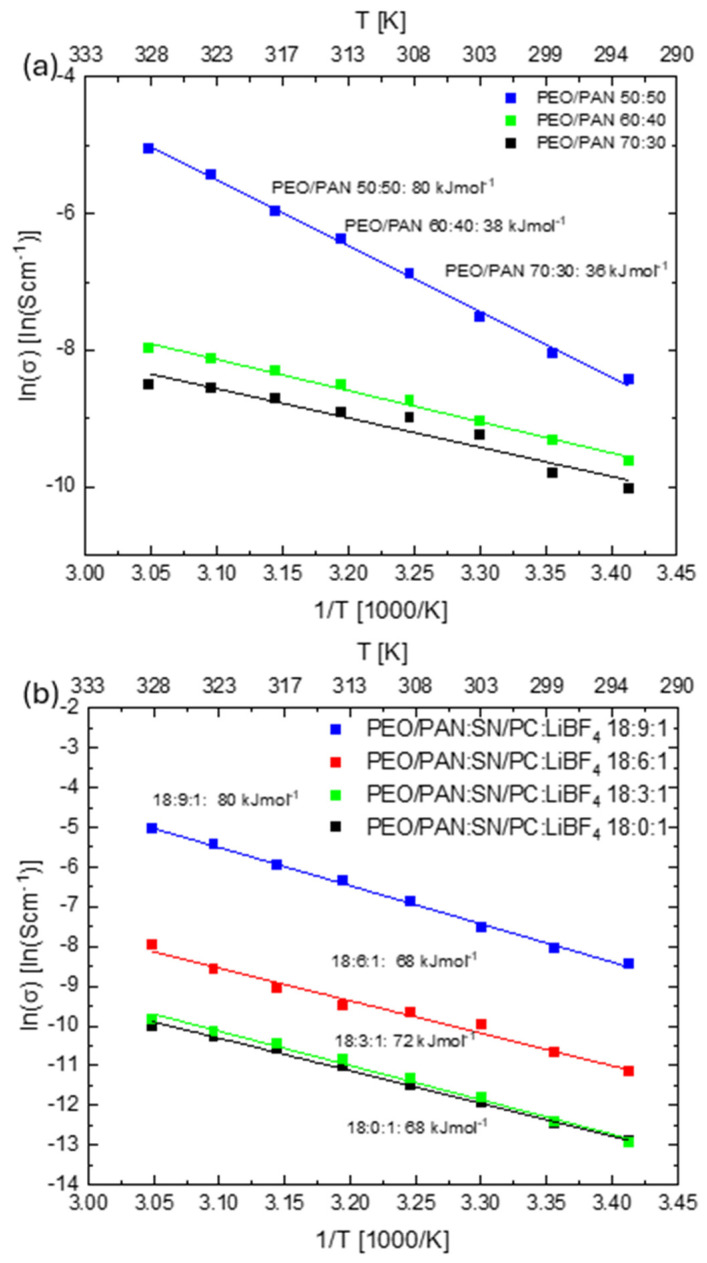
(**a**) Overview of the Arrhenius behavior of PEO/PAN systems with different PAN ratios. The composition with 50:50 shows the highest activation energy as well as the highest ionic conductivity. (**b**) Activation energies for PAN/PEO 50:50 (18:X:1) systems with varying plasticizer amounts X = 0, 3, 6, and 9.

Taking a closer look at the activation energies for the PEO/PAN systems, we intended to verify the effect of the PAN incorporation on ion coordination and mobility. An Arrhenius plot is given in Figure 2. The PEO/PAN 60:40 and 70:30 samples show activation energies of 38 and 36 kJ/mol, respectively, which are comparable to the high plasticizer containing pure PEO:SN:LiBF_4_ membranes (31 to 35 kJ/mol) reported in the literature [36]. Here, the PEO part seems to be the dominant fraction in determining the ion transport and conductivity. The 50:50 sample is somehow different because it displays the highest thermal activation among all, with an activation energy of 80 kJ/mol. Despite its high ionic conductivity of 1∙10^−4^ S/cm at room temperature and 1∙10^−2^ S/cm at 55 °C, the high activation energy is rather unexpected and cannot be explained or related to its PEO content alone. We believe that this increase in activation energy is related to the high surface area between the PEO and PAN fibers, where the PEO surface acts as the origin of ion conduction (higher conductivity than for the other PEO/PAN compositions) and the close vicinity of PAN somehow influences the thermal activation within the PEO. This aspect is subject to ongoing research.

Going to other plasticizers containing PEO/PAN (50:50) systems than the X = 9 one, we determined a rather comparable activation barrier. Upon reduction of the plasticizer contents to X = 6, 3, and 0, we found activation energies in the range of 70 kJ/mol. The plasticizer content has no significant influence on ion activation. Pure PEO systems with comparable composition also show activation barriers in the same range.

In the following, we intend to examine the polymer electrolyte properties closely to evaluate the properties of the PEO/PAN systems.

**Figure 3:**
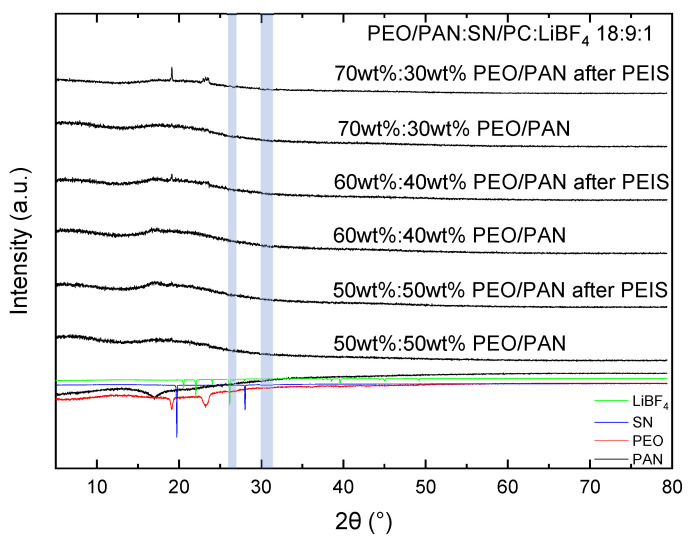
Overview of the XRD data of (18:9:1) systems with different PEO/PAN ratios. With high PEO content, a certain tendency for PEO crystallization is detected after PEIS measurements for the 60:40 and 70:30 samples. The 50:50 wt% composition shows no significant reflections either before or after electrochemical treatment. Intensities of measured pure PEO and PAN samples, as well as calculated LiBF_4_ and SN diffractograms, are drawn at the bottom with negative intensities for better comparison.

Since the conductivity of conductive salt containing PEO is known to be dependent on the grade of crystallinity, this feature was investigated using XRD [37]. Usually, the more amorphous the system is, the higher the conductivity will be. Figure 3 gives an overview of the X-ray diffractograms of the PEO/PAN (18:9:1) systems containing the largest amount of plasticizer. All other investigated systems are illustrated in the supplement section (Figure S2). We have conducted XRD diffractograms before and after the PEIS measurements in order to document the influence of the electrochemical treatment on the crystallinity of the investigated samples. All samples are non-crystalline prior to utilization and PEIS measurements, illustrating the effect of the plasticizer in reducing crystallinity. Also, the PAN content seems not to affect the crystallization of PEO. All samples are rather comparable in PAN crystallinity before and after PEIS. No tendency to crystallize is visible for all samples. The 70:30 wt% and 60:40 composition exhibit the dominant reflections of PEO at 19° and 23° after potentiostatic electrochemical impedance spectroscopy (PEIS) measurements (Figure 3, marked in blue). The absence of a strong crystallization tendency after PEIS is an important finding which illustrates that the crystallinity is not seriously affected by PEIS measurements.

An XRD overview in Figure S2 shows that a decreasing amount of plasticizer enlarges the crystallinity tendency of the samples. A brief discussion is given in the supplement. The 18:9:1 composition shows the lowest crystallinity, indicating the best potential for high conductivity, since ionic transport in PEO takes place in the amorphous regions of the electrolyte [27]. Bruce et al. reported that the ion transport in PEO happens in the amorphous phase, so a decrease in crystallinity is expected to result in a higher conductivity [37]. The observed trend of decreasing crystallinity with increasing amount of plasticizer, leading to a high conductivity, agrees with the conductivity measurements shown in Figure 1. The highest conductivity was determined for the 18:9:1 sample, and the lowest for the 18:3:1 and 18:0:1 samples, precisely as would be expected from the XRD analysis.

Glass transition and melting temperatures are important properties of polymer electrolytes. Figure S1 shows the DSC data for all samples analyzed in this study, including those with varied PEO fractions and those with varying amounts of plasticizer, which will be discussed further in this work. The melting points of all samples are rather similar, all in the temperature region from 42 to 56 °C, with no clearly discernible trend between the different compositions. Thus, the addition of higher amounts of PAN does not favorably affect the melting point of PEO, nor does it initiate crystallization.

The glass transition temperature follows the same pattern and can be detected at around −40 °C. A rather low glass transition point indicates an early mobility of the polymer chains, which in turn points towards a high conductivity at lower temperatures. It seems the case that the important PEO properties are not affected by PAN, which substantiates the innocence and matrix character of PAN.

**Figure 4:**
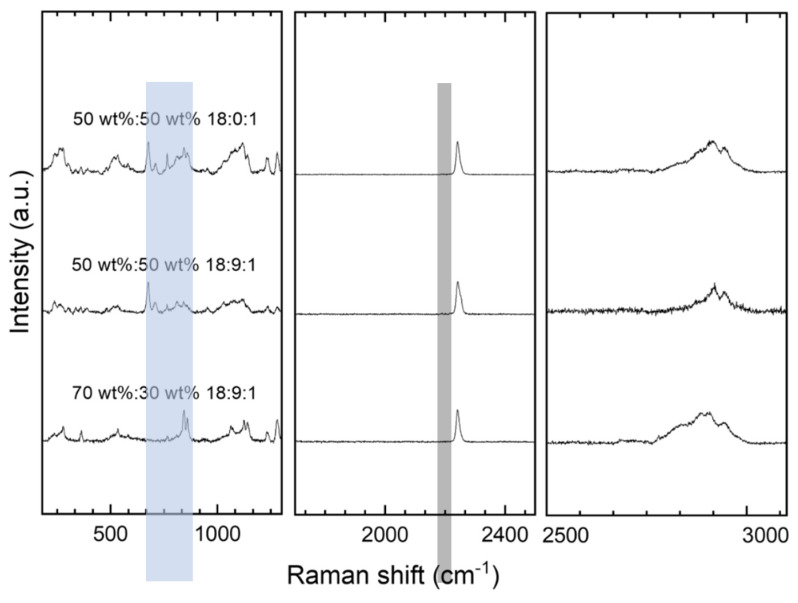
Raman analysis of selected PEO/PAN 50:50 (18:9:1) systems. Compositions with the highest (PEO/PAN 70 wt%:30 wt%) and the lowest amount of PEO (PEO/PAN 50 wt%:50 wt%), and a sample with no plasticizer were compared. Highlighted in blue is the significant PEO ether band at 850 cm^−1^ with different band broadening, which indicates the interaction with Li cations; Highlighted in grey is the classic PAN CN stretching vibration at 2250 cm^−1^.

Raman spectroscopy was employed to evaluate the molecular structure and crystallinity of the produced membranes. This analysis provided insights into the phase interactions between the polymer components and the local coordination environment of lithium ions. Results are shown in Figure 4. The left inset shows the CH group out-of-phase twisting modes, which occur at 1255 and 1239 cm^−1^. The stretching mode of the C-O-C at 1070 cm^−1^ arises from the polymer chain, but in contrast to the rocking mode at around 850 cm^−1^; this is not relevant for the lithium-ion conduction because it does not affect the chain motion. A C-O-C rocking mode at around 850 cm^−1^ can be found as a more defined band in the 70:30 sample, being an indicator of a slightly higher ordering tendency in PEO parts in the 70:30 membrane than in all other samples. This aspect fits the results from XRD analysis, at least for the situation after PEIS measurements (Figure 3). The position and the broadening of the bands were fitted with Lorentzian functions. A shift of the C-O-C rocking mode from 859 cm^−1^ for the 70:30 composition to 847 cm^−1^ for the 50:50 composition was observed. This band is sharper and more intense in the 70:30 than in the 50:50 PEO/PAN system; the latter is ~7 times broader. A lowered intensity of this band can be observed for the 50:50 composition, a phenomenon that is expected as a result of the beneficial and stronger lithium solvation effect on the vibrational modes of the PEO backbone [38]. The broadening of the characteristic PEO Raman band (highlighted blue in Figure 4. is indicative of a more heterogeneous environment around the polymer chains, which arises when lithium ions interact with PEO. In a well-ordered, crystalline PEO (not present in this study), vibrational modes are relatively sharp, resulting in rather sharp, defined Raman peaks. However, as lithium ions coordinate with the ether oxygens in PEO, they disrupt the regular polymer conformation and environment, which leads to variations in the vibrational frequencies of the polymer chains, thereby broadening the Raman mode. In essence, the broadened band reflects the increased disorder and certain ion–polymer interactions. The middle graph shows a characteristic CN stretching band (highlighted in grey in Figure 4) at around 2240 cm^−1^. The shape of this band is sharp and defined and signals that the CN group of the membrane is not actively involved in lithium-ion coordination and therefore also not in Li transport. PAN seems to act as a separating and stabilizing matrix for the PEO fraction. Furthermore, the bands at 2919 cm^−1^ are indexed to the presence of the CH and CH_2_ groups of the polymers and plasticizer. As they are chemically rather similar, it is not possible to distinguish between polymer and plasticizer.

Electrospinning as a method and the resulting morphology of the membranes were identified as an intriguing option for the improvement of the ion conductivity in PEO-based polymer electrolytes [33]. Due to this aspect, we investigated the morphology and fiber structures of the different systems in detail, especially to understand the conductivity improvement for the 50:50 in relation to PEO-richer systems.

**Figure 5:**
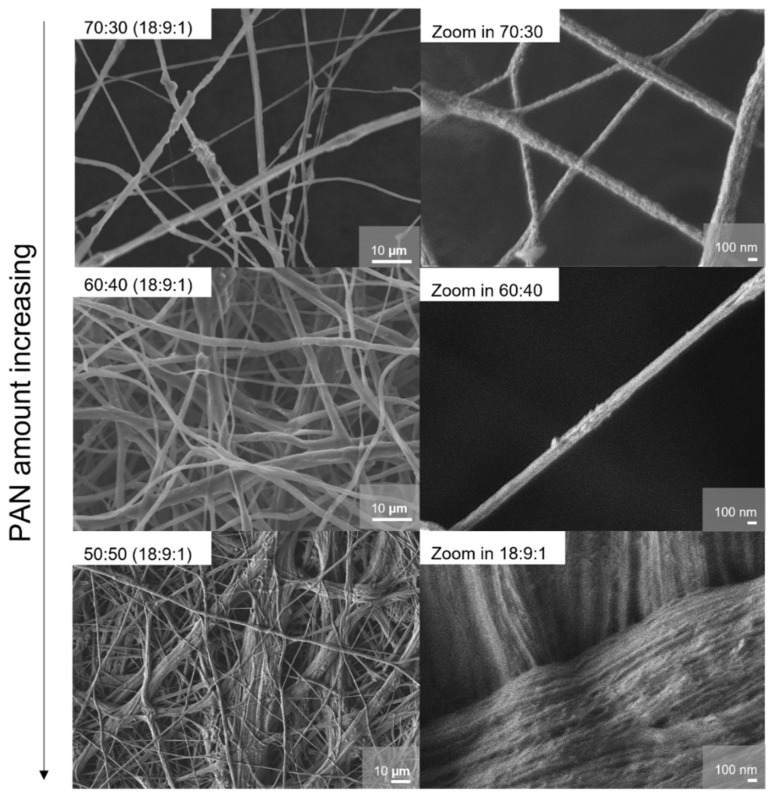
SEM Images of the composition PEO/PAN (18:9:1) systems with different PEO/PAN wt% ratios from 70:30, 60:40, and 50:50. The overall fiber structure changes with the addition of more PAN. Membranes with a low PAN amount have thin fibers and the membranes with a higher PAN amount show thicker, rope-like fibers consisting of many super-thin fibers.

**Figure 6:**
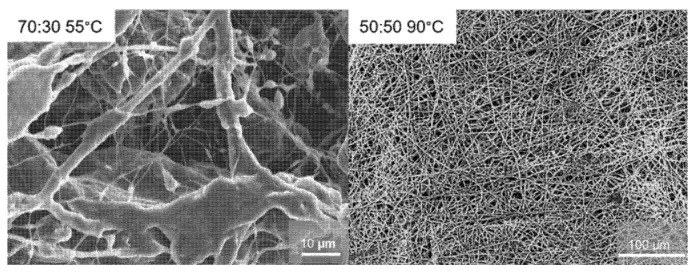
SEM Images of the PEO/PAN 70:30 (18:9:1) system heated up to 55 °C during the impedance measurement (**left**) and a PEO/PAN 50:50 (18:9:1) membrane after impedance spectroscopy and thermal treatment (heated up to 90 °C, **right**).

**Figure 7:**
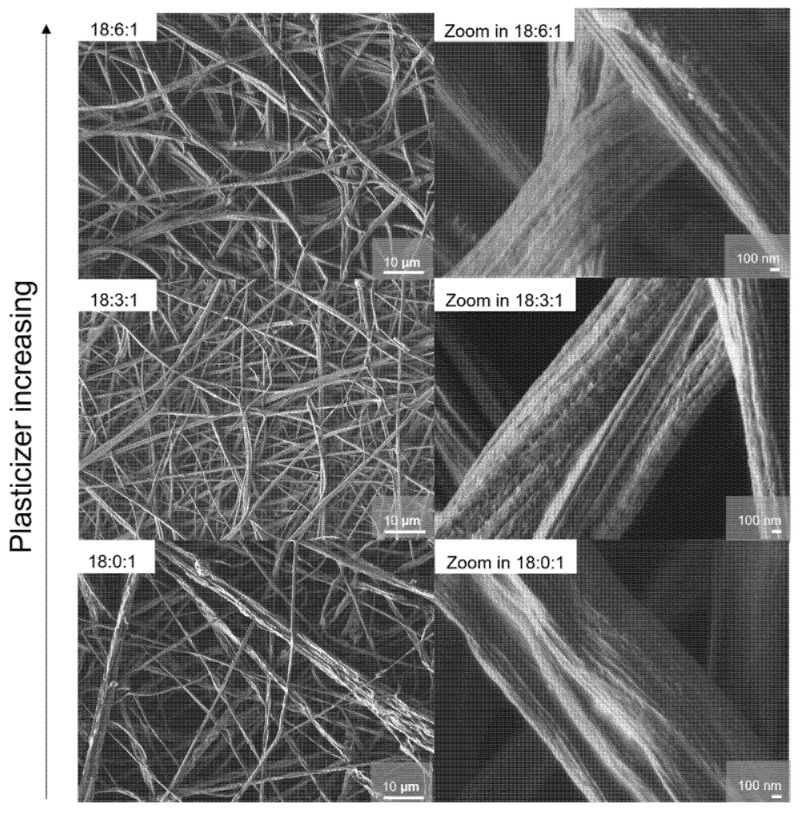
SEM images of the membranes with a varied plasticizer amount for PEO/PAN 50:50 (18 (PEO/PAN):X:1) systems with X = 0, 3, 6). All the structures show rope-like fiber bundles consisting of thinner fibers of around 40 nm.

The morphology of the samples was assessed via scanning electron microscopy; the results are shown in Figure 5–7. In principle, the fiber diameter is related and controlled by the ES synthesis parameters. In this study, parameters like spinning voltage, concentration of the polymer solution, feed rate, ES collector, and collector distance were kept constant. The appearance of the samples can be described by an overall hierarchical fiber structure, which leads to a densely arranged membrane. The fiber diameters lie mostly between 0.5 and 8 µm. The samples with higher PEO content (PEO/PAN 70:30, PEO/PAN 60:40) show a wider distribution of fiber diameters; the histograms of size distribution can be found in Figure S3 in the Supporting Information. Fibers thicker than 10 µm can be found in the 50:50 PEO/PAN system; they consist of various fiber bundles and with a rope-like hierarchical morphology. The fiber bundles consist of thinner fibers with a diameter of approximately 40 nm. We interpret this effect as a controlled phase segregation of PAN and PEO during ES. These slim structures generate a large surface area for both (phase segregated) systems, the PEO and PAN regions, with a very intricate connection between bundles. It has already been reported for electrospun PEO:LiBF_4_ membranes that an increase of the Li surface mobility vs. the bulk mobility is the origin of the conductivity increase in such systems. Due to the morphologic difference (that we assign to segregation), the PEO fiber diameter and therefore the surface-to-bulk ratio for the PEO fibers is significantly increased for the 50:50 sample compared with the other examples. We believe that this pronounced texturing creates areas with fast Li-ion mobility at the PEO fiber surface. The pronounced hierarchical morphology was not found in pure PAN membranes [39], nor in ES PEO/PAN (75:25) ones [40].

The morphology of the membranes after thermal treatment was also analyzed and shown in Figure 6. The difference between the PEO/PAN 70:30 and the 50:50 membranes is remarkable. The 70:30 membrane treated at 55 °C, close to the PEO melting temperature, shows large, melted regions with a low fiber count. On the other hand, the 50:50 membranes operated at 90 °C, well above the melting temperature of PEO, show almost no melted regions, and most of their initial structure (morphology) remains intact. A pure PEO membrane has fully lost its shape and morphology once heated to 90 °C, significantly above the melting point of PEO. This finding illustrates that PAN creates a stable scaffold for hosting PEO. The latter also seems not to be affected by its thermal properties as illustrated by DSC data (Figure S1). We found no significant shift either in the glass or the melting temperature of PEO for the three different systems. The intact texture and morphology provided in the 50:50 wt% sample would be an advantage in practical battery applications, given that batteries could be operated at temperatures higher than the PEO melting point, and possibly be more resistant against thermal overshoots without losing their primary function.

Based on these observations, a series of experiments were conducted to find an optimal PEO/PAN blend polymer membrane with a 50:50 weight ratio and to verify if the SN plasticizer composition may influence the segregation effect. The impact of the plasticizer was already studied in PEO-based SPEs, with an increase in conductivity through enhanced chain mobility, which in turn increased the ion mobility in the material [37]. The morphology of the samples prepared with varying amounts of plasticizer was assessed via SEM (Figure 7). For the 50:50 samples, the same hierarchical structure is observed regardless of the starting amount of plasticizer. Nevertheless, increasing amounts of SN lead to size distributions skewed towards larger diameters, as illustrated in the histograms in Figure S4 in the Supporting Information section. Samples (18:0:1) and (18:3:1) display fibers with diameters mostly below 10 µm, while membranes with higher plasticizer content show thicker structures.

The incorporation of PAN into PEO induces a phase segregation phenomenon due to their distinct solubility behaviors in the chosen solvent system. This segregation leads to the formation of well-defined microdomains and fiber bundles within the electrospun fibers, generating unique interfacial regions on the fiber surface that serve as additional ionic pathways. A schematic view of the interconnection between PEO and PAN fibers is given in the supplement. Due to enhanced ion mobility and conductivity at the PEO surface caused by different surface coordination of the Li-ions compared with bulk ions within the fibers, one achieves ion conduction in all directions parallel and perpendicular to the membrane (Supplementary Section and Figure S5). These interfaces facilitate enhanced ion mobility and contribute to the overall ionic conductivity by providing alternative routes for lithium-ion transport, which are not available in homogeneous polymer matrices. Consequently, the synergistic interaction between PAN and PEO in the electrospun membrane not only leverages their individual properties but also capitalizes on the beneficial effects of phase segregation, promoting superior conductivity and performance in solid polymer electrolyte systems.

**Figure 8:**
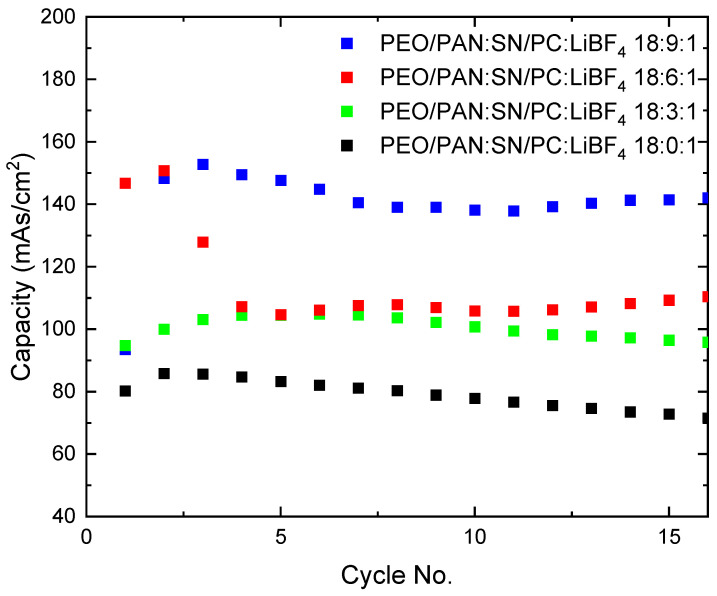
Symmetrical capacity overview of coin cells built with different compositions.

One aspect besides exhibiting high ionic conductivity for solid electrolytes in secondary battery systems is the stability against metallic lithium. In order to assess this character, cyclic voltammetry was performed with a symmetric cell setup (Li|(PEO/PAN):SN/PC:LiBF_4_|Li). Cyclic voltammetry (CV) curves (Supplementary Figure S6) and the corresponding capacity versus cycle number (Figure 8) demonstrate reversible lithium-ion transport through the electrospun membranes for up to 16 consecutive cycles. All systems started to fail after 16 cycles, while the plasticizer-free sample remained intact for up to 40 cycles. This fading is most probably due to the porous membrane architecture rather than any material properties that need to be improved for battery applications.

The sample with the highest nominal plasticizer content—the 18:9:1 system—demonstrated the best capacity performance, reaching 140 mAs/cm^2^ in symmetrical lithium–lithium cells. This value aligns well with the upper range reported in the literature for PEO-based SPEs under similar conditions, where typical areal capacities range from approximately 100 to 200 mAs/cm^2^, depending on the formulation and testing temperature. For instance, Liu et al. reported stable lithium symmetric cycling using plastic crystal–polymer composite electrolytes, while Holmes et al. showed how electrolyte composition directly influences cycling stability in symmetric cells [41,42]. These comparisons suggest that the electrochemical performance of the 18:9:1 system is competitive with non-porous membranes. In the first test, we assembled a Li|PEO/PAN 18:9:1|lithium iron phosphate (LFP) cell and tested the ability to use the SPE in a battery environment. The 18:9:1 system can be operated up to 4.1 V in the present setup. Data are summarized in the supplement (Figure S7).

In order to put this entire study in a broader SPE context, it is important to compare properties (e.g., segregation tendency or ion conductivity) of the phase-segregated PEO/PAN SPEs with already reported systems. First, the ratio of PEO to PAN seems important because higher PEO contents than (50:50) obviously do not show this pronounced segregation effect that leads to separated fiber bundles. Abdollahi et al. reported on an ES (75:25) system, which did not show this visible segregation within the ES polymer fibers to fiber bundles, but they noted that miscibility and phase separation were indicated even for this system [40].

If one intends to verify the ion conductivity of the title systems in relation to known polymer electrolytes, one first has to differentiate gel (GPE), solid (SPE), and composite polymer electrolytes (CPEs). GPEs contain a polymer matrix swollen in liquid electrolytes. SPEs are “dry” solvent-free systems without organic or ionic liquids where ion transport is realized via the solid phase. CPEs are systems using techniques like polymer blending, cross-linking polymer matrices, binary salt systems, incorporation of additives, doping of nanomaterials, impregnation with ionic liquids, or reinforcement by inorganic fillers to overcome drawbacks from aforementioned systems. Nice review articles reflect this issue [18,43].

In the supplement, we summarized the conductivities of various ES polymer electrolyte systems using PEO and PAN in various compositions and forms (Figure S8). Due to the manifold variations and systems possible, only a small number of systems are denoted in Table S1. Many of them are GPEs [40,44,45,46,47], one intriguing recent report deals with an ES PAN membrane that is encapsulated in liquid electrolyte-activated PEO [44], or even complex multilayer and multi-copolymer GPE systems [45]. In such systems, the room temperature conductivities range from approx. 5∙10^−3^ to 10^−4^ S/cm. ES- and casted PEO CPEs with various plasticizer additives, inorganic fillers, and conductive salts reach conductivities most frequently between 10^−4^ and 10^−5^ S/cm [44]. If only conductive salt is added, pure ES-PEO/LiBF_4_ SPE membranes show ion conductivities of 1.5∙10^−6^ S/cm. An increase in the chain mobility by SN pushed the ion conductivity to 2∙10^−4^ S/cm. Pure ES- and casted PAN show no significant conductivity. The phase segregated PEO/PAN (50:50) 18:9:1 system featuring a conductivity of 1.0∙10^−4^ S/cm show almost no difference from the pure PEO systems. Furthermore, in the supplement Figure S9 shows the general comparison of the PEO/PAN electrospun system with other solid electrolytes.

Taken together, these results highlight the importance of carefully balancing ionic conductivity, mechanical integrity, and electrochemical durability to further optimize solid polymer electrolytes for advanced lithium-metal battery applications.

It is to mention at this stage that the handling and also ES fabrication of the membranes are best for high-plasticizer-containing systems. A drawback of ES is the evaporation of the plasticizer during the ES workup procedure, which cannot be avoided. This aspect needs to be taken into account if the ES material is compared with other (HP or SC) fabricated materials.

## 4. Conclusions

This study illustrates the advantages of incorporating thermally stable PAN into ion-conducting PEO. Various PAN/PEO ratios were studied to identify the conductivity optimum and the composition with the most promising thermal and electrochemical characteristics. Among others, the 50:50 composition exhibited the highest conductivity values (approaching 10^−2^ S/cm at ~50 °C and 10^−4^ S/cm at r.t.). SEM analysis revealed that this sample showed phase segregation and contained two different fiber morphologies: standalone fibers and rope-like structures composed of thinner fibers (~40 nm). These distinct morphologies are proposed to be responsible for the creation of new lithium-ion pathways in this electrolyte.

The influence of SN content on the 50:50 membranes was also examined. Samples with lower SN content displayed fewer, larger fibers (>10 µm) and lower conductivities, suggesting insufficient chain mobility and less efficient ion transport.

The 18:9:1 membrane, which represents an optimized balance of plasticizer and polymer composition, achieved the highest capacity (~140 mAs/cm^2^) in symmetrical cells. The results indicate that the interplay between plasticizer content, phase separation, and fiber morphology is crucial to achieving the desired balance between conductivity, stability, and processability.

In sum, PAN can be effectively added to PEO SPEs without affecting the overall Li conductivity of the system. A PEO/PAN 50:50 wt% ratio and a high SN content of X = 9 in a (18:X:1) system were identified as the most powerful and optimized material. Between no(low)-plasticizer and high-plasticizer-containing systems, the conductivity can be enlarged by about two orders of magnitude.

Overall, this study demonstrated the potential of electrospun and phase-segregated polymer blends for effective polymer electrolytes.

## Figures and Tables

**Table 1:** Starting material amounts for the synthesis of each polymer system. The weight fraction of PEO/PAN and the amount of plasticizer have been varied. Starting composition (e.g., 18:9:1) is given as a molar ratio based on the respective repetitive polymer unit, M_PEO_ = 44.03 g/mol, M_PAN_ = 53.06 g/mol, M_LiBF4_ = 93.75 g/mol, M_SN_ = 80.03 g/mol, M_PC_ = 102.09 g/mol. For the calculation of the compositions, the molar ratios were calculated separately; PEO:SN:LiBF_4_ and PAN:PC:LiBF_4_ were calculated in the molar ratios of 18:9:1. The polymers were taken in wt% ratios and mixed as described. After the calculation, the polymer composition was treated as one system.

Polymer Ratio in wt% (Polymer: Plasticizer:LiBF_4_ Molar Ratio in 18:X:1 with Varying Plasticizer Content)	PEO (g)	PAN (g)	SN (g)	PC (g)	LiBF_4_ (g)	DMSO (mL)	MeCN (mL)
PEO/PAN 70:30 (18:9:1)	0.3500	0.1500	0.3183	0.1443	0.0561	6.0	6.0
PEO/PAN 60:40 (18:9:1)	0.3600	0.2400	0.3274	0.2309	0.0661	6.2	6.2
PEO/PAN 50:50 (18:9:1)	0.2500	0.2500	0.2274	0.2405	0.0541	4.5	4.5
PEO/PAN 50:50 (18:6:1)	0.2500	0.2500	0.1516	0.1326	0.0541	4.5	4.5
PEO/PAN 50:50 (18:3:1)	0.2500	0.2500	0.0758	0.0802	0.0541	3.0	3.0
PEO/PAN 50:50 (18:0:1)	0.2500	0.2500	0	0	0.0541	3.0	3.0

## Data Availability

The raw data supporting the conclusions of this article will be made available by the authors on request.

## Supplementary Materials

The following supporting information can be downloaded at: https://www.mdpi.com/article/10.3390/membranes15070196/s1, Figure S1. Overview of DSC measurements of the PEO/PAN samples; Figure S2. XRD analysis of the samples with varying plasticizer amount; Figure S3. Overview of the histograms of fiber diameter for PEO/PAN (18:9:1) membranes with varying PAN fractions based on the SEM images. Figure S4. Overview of the histograms of fiber diameter for PEO/PAN (18:X:1) membranes (X = 0, 3, 6) with varying plasticizer fractions based on the SEM images. Figure S5. Top part: Illustration of the coordination behavior of ions in an ES polymer fiber; Figure S6. CV curves of the samples 18:9:1 and 18:0:1 (polymer blend:plasticizer:conductive salt additive); Table S1. Summary of various polymer electrolytes. Figure S7. CV of an Li|PEO/PAN (18:9:1)|LFP cell cycled around the open circuit potential with a rate of 0.1 mV/s; Figure S8. Nyquist-plots at room temperature are denoted normalized on the membrane thickness. Figure S9. This graphic shows the comparison of other electrolyte classes with the electrolyte systems in this study. References [12,13,14,15,23,33,35,36,40,43,44,45,46,47,48,49,50] are citations that are used and discussed also in the Supplementary Materials.
