# Supplementary material for: Highly Conductive PEO/PAN-Based SN-Containing Electrospun Membranes as Solid Polymer Electrolytes
Source: Membranes (Basel). 2025 Jun 30;15(7):196. doi: 10.3390/membranes15070196 (PMC12300085; doi:10.3390/membranes15070196)
Supplement: Supplementary file 1 [file membranes-15-00196-s001.zip › membranes-3655496-supplementary.pdf]

membranes

Supplementary Materials

Highly Conductive PEO/PAN-Based SN-Containing Electrospun Membranes as Solid Polymer Electrolytes

Anna Maria Kirchberger [1,2], Patrick Walke [1,2], Janio Venturini [1], Leo van Wüllen [3] and Tom Nilges [1,*]

1 TUM School of Natural Sciences, Technische Universität München, Lichtenbergstr. 4, 85748 Garching bei München, Germany
2 TUMint.Energy Research GmbH, Lichtenbergstr. 4, 85748 Garching bei München, Germany
3 Institute of Physics, University of Augsburg, Universitätsstraße 1, 86159 Augsburg, Germany
* Correspondence: tom.nilges@tum.de

Thermal analysis of PEO/PAN:plasticizer:conductive salt systems

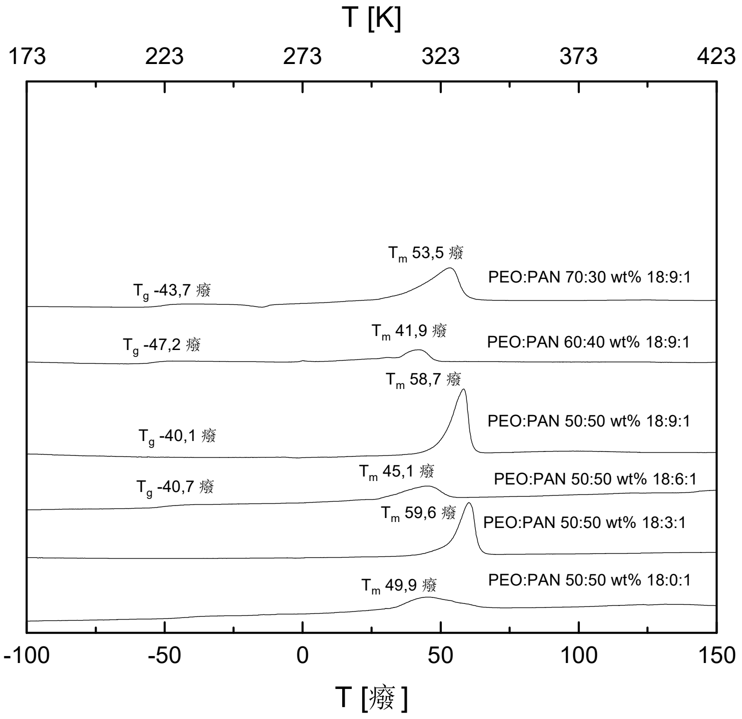

Figure S1. Overview of DSC measurements of the PEO/PAN samples with various PEO/PAN and SN ratio (polymer blend:plasticizer:conductive salt additive in wt%). The samples with varying plasticizer and constant PEO/PAN amount are also shown here. No clear trend between the different systems can be observed.

 https://doi.org/10.3390/membranes15070196

Powder XRD analysis of PEO/PAN:plasticizer:conductive salt systems

An XRD overview monitoring the crystallinity of the polymer electrolyte systems is given in Figure S2. An decreasing amount of plasticizer enlarges the crystallinity tendency of the samples. This phenomenon can be observed in the samples with composition 18:0:1 and 18:3:1 (which contain a rather low plasticizer amount), with intense reflections at 19° and 23° related to the presence of crystalline PEO. The diffractogram of the 18:0:1 sample show dominant reflections of PEO before and after the potentiostatic impedance measurement. The 18:3:1 composition show the reflection after applying voltage during the impedance measurement, which we believe is due to a beginning nucleation driven either by temperature, the electrochemical treatment or both. The 18:6:1 composition shows a weak PEO reflection at 19°, albeit reduced significantly compared to the 18:3:1 case.

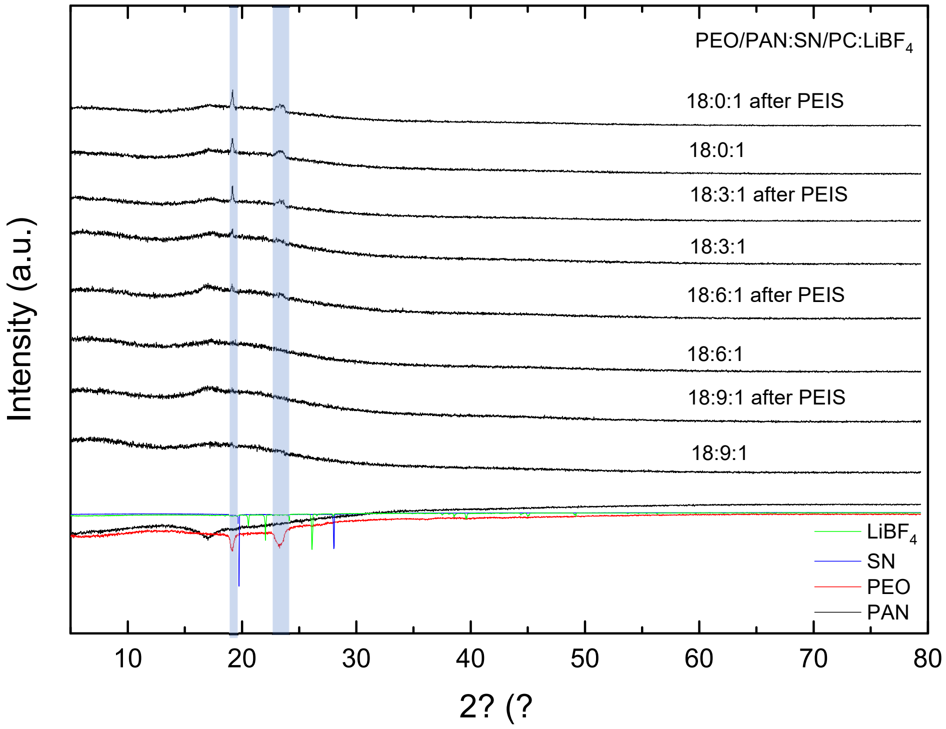

Figure S2. XRD analysis of the samples with varying plasticizer amount. Reflections present in non-SN and low-SN containing PEO/PAN systems are vanishing upon SN content increase. A decrease of crystallinity with increasing plasticizer amount can be observed top down. Reflections of partially crystalline PEO are marked in blue.

Histograms of PEO/PAN:plasticizer:conductive salt systems

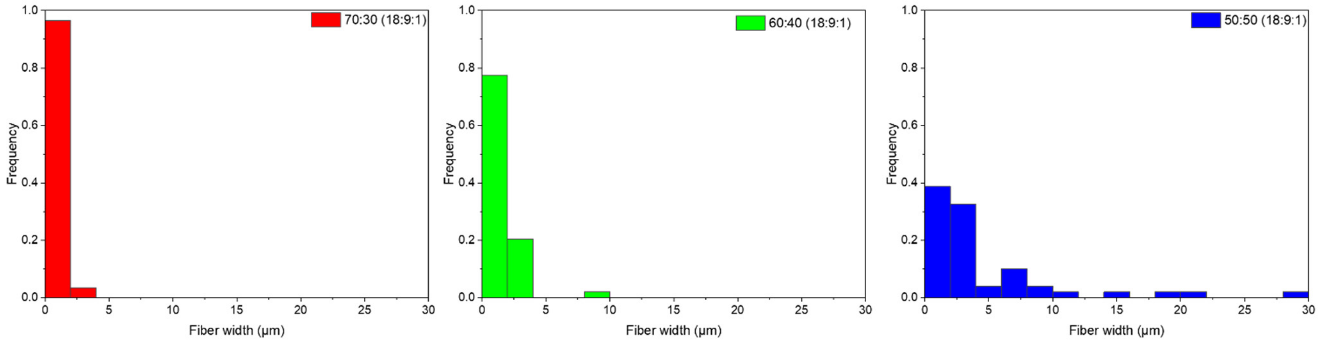

Figure S3. Overview of the histograms of fiber diameter for PEO/PAN (18:9:1) membranes with varying PAN fractions based on the SEM images.

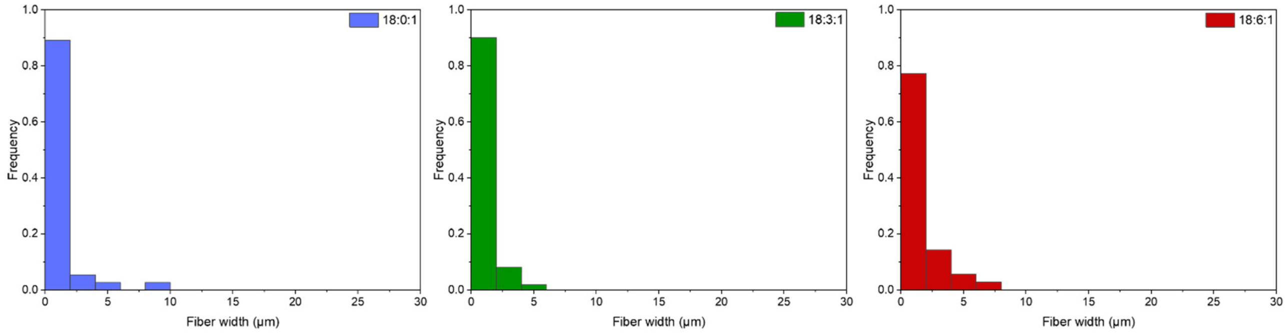

Figure S4. Overview of the histograms of fiber diameter for PEO/PAN (18:X:1) membranes (X = 0, 3, 6) with varying plasticizer fractions based on the SEM images.

Conduction mechanism model for ion transport in phase segregated PEO/PAN membranes

Lithium ions can be transported through PEO/PAN membranes, as substantiated by conductivity measurements and symmetric CV measurements between Li metal. In previous literature, the local mobility in electrospun pure PEO membranes was evaluated and has been monitored by NMR spectroscopy [33,35,36]. Here, a high ion mobility was found for such PEO membranes. The high ion conductivity in the ES fiber membranes (overcoming cased membranes by up to two orders of magnitude) was connected to the fiber architecture and a pronounced and accelerated mobility of the Li ions at the surface vs. bulk ones (see Figure S5, top part). For PAN- based SPEs to opposite is true, PAN did not show any ion conducting properties with $LiBF_4$ as conductive salt additive. Based on these findings and taking all other variations of this study into account we suggest a possible ion conduction mechanism for phase segregated PEO/PAN (50:50) systems. Due to the random distribution of PEO and PAN fibers reasonable PEO contacts occur in the ES membrane that allows ion transport parallel and perpendicular to the fibers (Figure S5, bottom part).

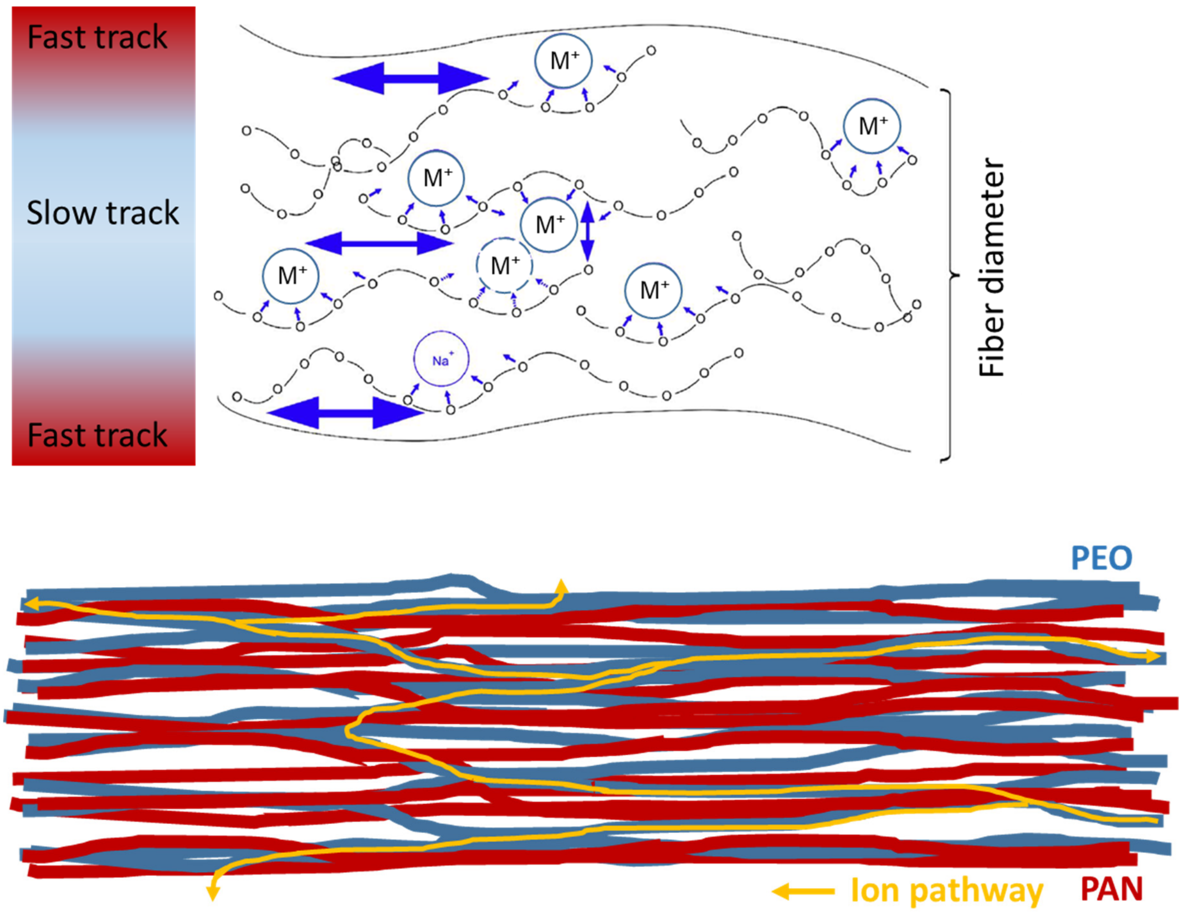

Figure S5. Top part: Illustration of the coordination behavior of ions in an ES polymer fiber. Due to different coordination behavior of surface ions vs. bulk ions within the fiber an enhanced ion movement and transport occurs in the ES fibers. Once such fibers are in contact with each other a transport of ions perpendicular to the fiber direction is also possible.
Bottom part: The illustration shows an arrangement of PEO (blue) and PAN (red) fibers aligned in one direction with random distribution and a random but pre-oriented alignment along a given direction. Each fiber bundle has significant contact with either the same and the opposite polymer in an random fashion. Taking a pronounced and also accelerated mobility of Li-ions on the surface of PEO fibers into account, an ion movement along and perpendicular to the membrane is possible.

Cyclic voltametry (CV) of PEO/PAN:plasticizer:conductive

The curve of the 18:9:1 membrane (in blue) in Figure S 6 has its current maximum between 0.08 mA and 0.1 mA and shows a wide area under the curve with a characteristic shape. Data from composition 18:0:1 (black curve) shows the maximum current between 0.03 mA and 0.05 mA. This curve is symmetric, but its shape is more compressed than 18:9:1. The capacities and the CV curves show a quick formation process in the initial cycles, which stabilizes after 3-10 cycles. The capacity of the 18:9:1 composition lies at around 140 As/cm², which is 60 As/cm² above the initial capacity of the sample with no plasticizer.

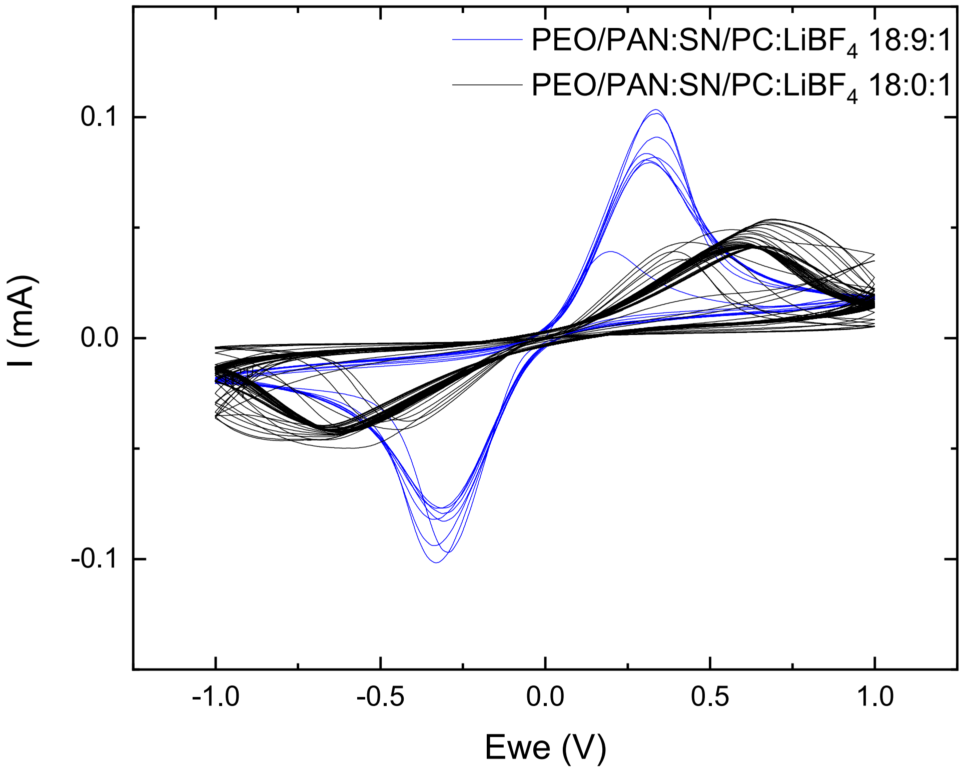

Figure S6. The graphic on the left side shows the CV curves of the samples 18:9:1 and 18:0:1 (polymer blend:plasticizer:conductive salt additive).

Table S1 gives an overview on selected electrosun GPEs, CPEs and SPEs, using PEO and PAN in various ratios and with different additives. Comments are denoted to illustrate the differences of the systems. Conductivities are given at room temperature unless otherwise stated.

Table S1. Summary of various polymer electrolytes.

System	Conductivity [S/cm]	Comments	Type of electrolyte	Lit.
ES-PEO/PAN (75:25 wt%)	$3.7 \cdot 10^{-3}$	Electrospun membranes were soaked in 0.5M LiI, 0.05M I_2 in PC/DEC (1:1, v/v)	GPE	[40]
ES-PEO/PAN (75:25 wt%)	$3.6 \cdot 10^{-3}$	0.5 M LiI, 0.05 M I_2 in PC/DEC (1:1, v/v)	GPE	[47]
ES-PAN fibers encapsulated by solution casted PEO	$1.2 \cdot 10^{-3}$	PEO was boosted with the liquid $LiPF_6$	CPE	[44]
ES-multilayer PAN/PEO system,	$4.72 \cdot 10^{-3}$	Multilayer composite polymer electrolyte, electrospun polyacrylonitrile (PAN) and inner layer of poly(vinyl acetate) (PVAc)/poly(methyl methacrylate) (PMMA)/poly(ethylene oxide) (PEO) fibrous membrane. Liquid electrolyte (1 M $LiPF_6$ in ethylene carbonate/dimethyl carbonate)	GPE	[45]
PEO-LiTFSI-IL	~ $3 \cdot 10^{-4}$	various ionic liquids (IL)	GPE	[46]
PEO SPEs with inorganic fillers like TiO_2, SiO_2, Al_2O_3, SnO_2, ZnO	10^{-4} to 10^{-5}	PEO as base polymer with various fillers and additives like EC, DEC, PVDF, LLTO, LLZAO and many others	CPE, dry, solid electrolyte	[43]
ES-PEO/sodium alginate (SA) nanofiber	$6.82 \cdot 10^{-5}$ at 303 K	LiTFSI, PEGdMA, LLZTO, AIBN, and sodium alginate (SA) nanofiber additives	CPE	[48]
ES-PEO	$1.5 \cdot 10^{-6}$	only $LiBF_4$ as conductive salt additive	SPE	[36]
ES-PEO/SN	$2 \cdot 10^{-5}$	$LiBF_4$ and Succinonitrile (SN) (18:3:1)	SPE	[36]
ES-PEO/SN	$2 \cdot 10^{-4}$	$LiBF_4$ and Succinonitrile (SN) (36:8:1)	SPE	[36]
ES-PAN	no ion conductivity	No coordination of cations within the PAN polymer.	n.a.	[33]
ES PEO/PAN (50:50)	$1.0 \cdot 10^{-4}$	$LiBF_4$ and Succinonitrile (SN) (18:9:1)	SPE	this study

Lithium iron phosphate (LFP)│SPE│Li cell testing

Experimental

The LFP cathode mixture was prepared in a glass vessel. The binder was added in the ratios of the used electrolyte in the measurements (e.g. if the composition 18:6:1 was measured, the binder recapture is also in the ratio 18:6:1). The mixture consists of 60 % LFP and 30 % C65 bond with 10 % polymer binder in a certain ratio. N-Methyl-2-pyrrolidon was used to solve all components. The cathode was prepared via solution casting. After production the electrode was dried on the Schlenkline. Full cells were prepared following the routine as stated below, and are cycled against the open

circuit potential between -1 and 1 V with a rate of 0.1 mV at r.t. The cells contain lithium metal electrodes with a diameter of 14 mm and membranes with a diameter of 17 mm, the LFP cathodes are test materials which were not optimized for this application, with a diameter of 14 mm. All cells were prepared in the glovebox under argon atmosphere (O_2 < 0.1 ppm; H_2O < 0.1 ppm). No additional additives or electrolytes were used.

First tests were conducted against Li metal and LFP as electrodes (Figure S8) to verify the stability of the SPE at high potentials. In a Li ∣ polymer ∣ LFP configuration the cyclic voltammetry illustrates that potentials up to 4.1 V are possible. The CV clearly shows the expected redox peaks of the LFP cathode (~4.1 V for Li extraction and ~3.4–3.5 V for Li insertion), while no additional irreversible reactions or significant parasitic currents were observed outside this range. This demonstrates that the polymer membrane remains electrochemically stable throughout the potential window relevant to typical Li-ion battery operation. Overall, the combination of Li ∣ Li+ and LFP ∣ Li measurements confirms that the electrospun PAN/PEO membrane provides an electrochemically stable environment across the voltage range explored in this study. It has to be stated that full cathode testing is currently underway. It is important to note that conventional cell preparation methods, such as the pressing step prior to cycling, tend to compromise key characteristics of the electrospun membrane. Therefore, in the present study, we used an unoptimized cathode and cycling method solely to demonstrate the electrochemical stability window of the material.

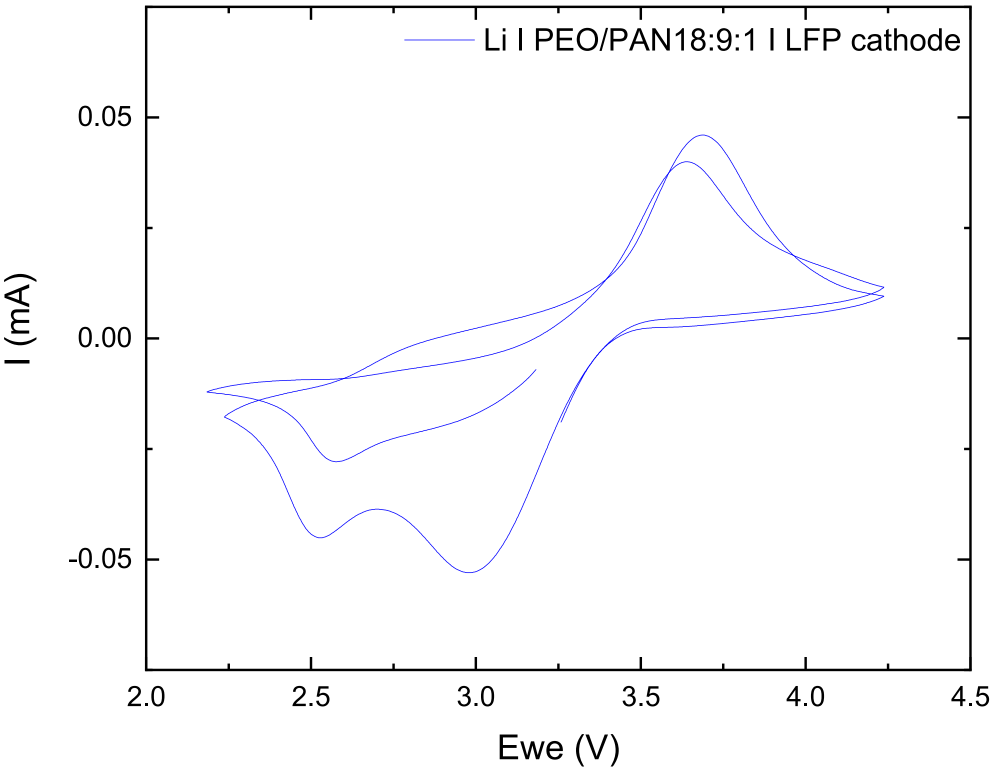

Figure S7. CV of an Li∣PEO/PAN (18:9:1)∣LFP cell cycled around the open circuit potential with a rate of 0.1 mV/s.

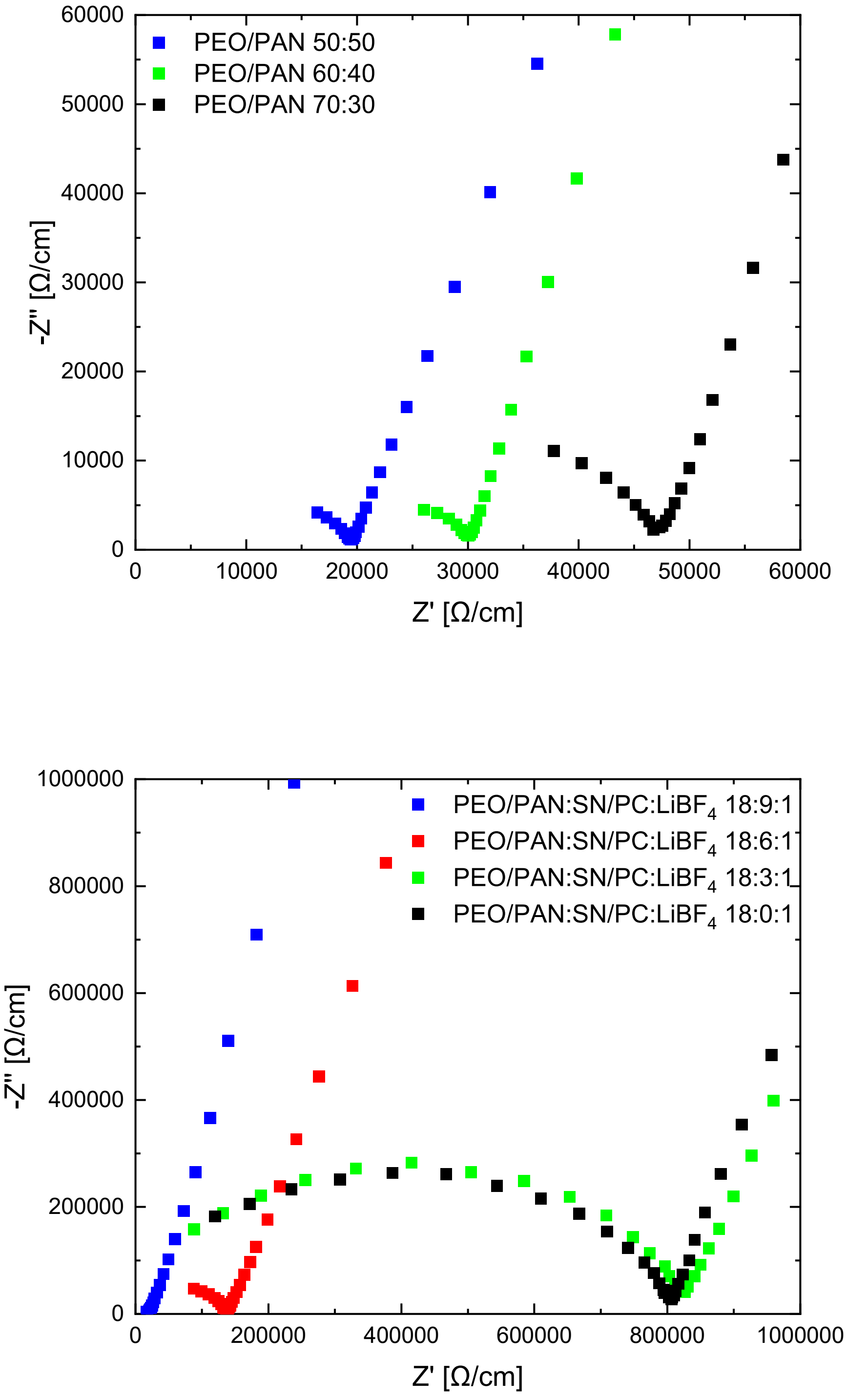

Figure S8. Nyquist-plots at room temperature are denoted normalized on the membrane thickness.

Conductivities of ES-PEO/PAN systems

In this section, the ionic conductivity of the studied electrolyte is compared first with representative values from other common classes of solid electrolytes, including oxide-, superionix-, sulfide-, and halide-based systems. This broader comparison displayed in Figure S7. highlights the general performance range and places the material in context with established benchmarks. Subsequently, the conductivity is specifically evaluated in relation to other polymer electrolytes and electrospun electrolytes reported in the literature, providing a more focused perspective on its competitiveness within this subclass of solid-state electrolytes.

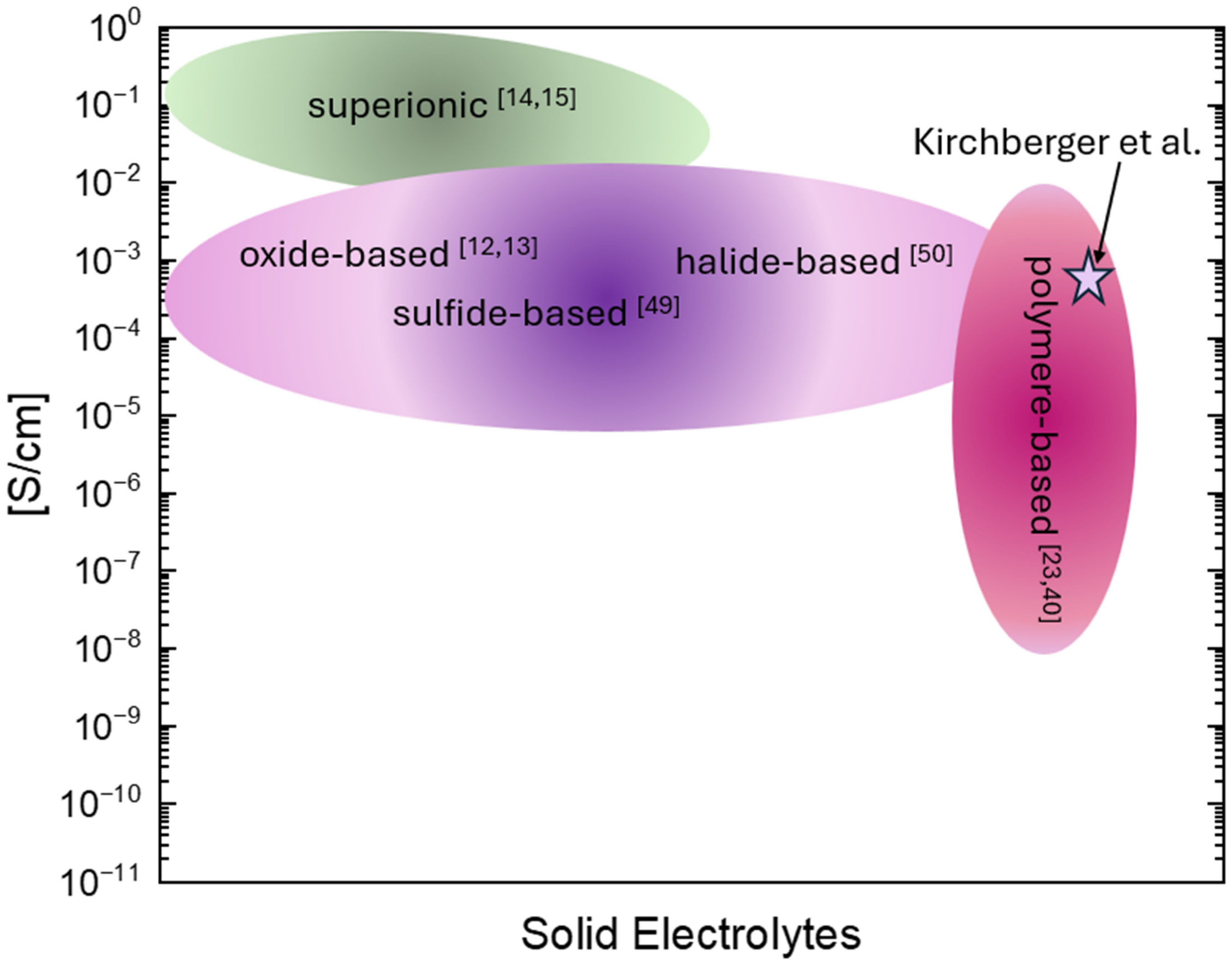

Figure S9. This graphic shows the comparison of other electrolyte classes with the electrolyte systems in this study. Here it is visible that the studied electrospun electrolytes are compatible within the polymer electrolyte systems.

References

12. Chen, L.; Li, Y.; Li, S.-P.; Fan, L.-Z.; Nan, C.-W.; Goodenough, J.B. Peo/garnet composite electrolytes for solid-state lithium batteries: From "ceramic-in-polymer" to "polymer-in-ceramic". Nano Energy 2018, 46, 176–184. https://doi.org/10.1016/j.nanoen.2017.12.037.
13. Thangadurai, V.; Kaack, H.; Weppner, W.J. Novel fast lithium ion conduction in garnet-type Li5La3M2O12 (m= Nb, Ta). J. Am. Ceram. Soc. 2003, 86, 437–440. https://doi.org/10.1111/j.1151-2916.2003.tb03318.x.
14. Kanno, R.; Murayama, M. Lithium ionic conductor thio-lisicon: The Li2S GeS2 P2S5 system. J. Electrochem. Soc. 2001, 148, A742. https://doi.org/10.1149/1.1379028.
15. Suzuki, K.; Kato, D.; Hara, K.; Hirayama, M.; Hara, M.; Kanno, R. Composite sulfur electrode for all-solid-state lithium–sulfur battery with Li2S–GeS2–P2S5-based thio-lisicon solid electrolyte. Electrochemistry 2018, 86, 1–5. https://doi.org/10.5796/electrochemistry.17-00055.
23. Croce, F.; Appetecchi, G.B.; Persi, L.; Scrosati, B. Nanocomposite polymer electrolytes for lithium batteries. Nature 1998, 394, 456–458. https://doi.org/10.1038/28818.
33. Voigt, N.; van Wüllen, L. The mechanism of ionic transport in PAN-based solid polymer electrolytes. Solid State Ion. 2012, 208, 8–16. https://doi.org/10.1016/j.ssi.2011.11.031.
35. Spranger, R.J.; Leo, W.; Kirchberger, A.; Nilges, T. Submitted: Highly-conductive mixed PEO/PAN-based membranes for solid state Li-ion batteries via electro-spinning and hot-press synthesis routes. Z. Für Anorg. Und Allg. Chem. 2025, in review.
36. Freitag, K.M.; Kirchhain, H.; Wüllen, L.; Nilges, T. Enhancement of Li ion conductivity by electrospun polymer fibers and direct fabrication of solvent-free separator membranes for Li ion batteries. Inorg. Chem. 2017, 56, 2100–2107. https://doi.org/10.1021/acs.inorgchem.6b02781.
40. Abdollahi, S.; Ehsani, M.; Morshedian, J.; Khonakdar, H.A.; Reuter, U. Structural and Electrochemical Properties of PEO/PAN Nanofibrous Blends: Prediction of Graphene Localization. Polym. Compos. 2018, 39, 3377–3816. https://doi.org/10.1002/pc.24390.
43. Li, X.; Deng, Y.; Li, K.; Yang, Z.; Hu, X.; Liu, Y.; Zhang, Z. Advancements in Performance Optimization of Electrospun Polyethylene Oxide-Based Solid-State Electrolytes for Lithium-Ion Batteries. Polymers 2023, 15, 3727. https://doi.org/10.3390/polym15183727.
44. Arifeen, W.U.; Akkinepally, B.; Abideen, Z.U.; Hussain, I.; Siddiqui, M.R.; Li, S.; Shim, J.; Ko, T.J. Electrospun PAN membrane encapsulated in PEO as a polymer electrolyte for lithium metal batteries. J. Ind. Eng. Chem. 2025, 142, 746–752. https://doi.org/10.1016/j.jiec.2024.11.054.
45. Lim, D.-H.; Haridas, A.K.; Figerez, S.P.; Raghavan, P.; Matic, A.; Ahn, J.-H. Tailor-Made Electrospun Multilayer Composite Polymer Electrolytes for High-Performance Lithium Polymer Batteries. J. Nanosci. Nanotechnol. 2018, 18, 6499–6505. https://doi.org/10.1166/jnn.2018.15689.
46. Pandey, G.P.; Kumar, Y.; Hashmi, S.A. Ionic liquid incorporated polymer electrolytes for supercapacitor application. Indian J. Chem. 2010, 49, 743–751.
47. Abdollahi, S.; Sadadi, H.; Ehsani, M.; Aram, E. Highly efficient polymer electrolyte based on electrospun PEO/PAN/single-layered graphene oxide. Ionics 2021, 27, 3477–3487. https://doi.org/10.1007/s11581-021-04105-x.
48. Sun, Q.; Liu, Z.; Zhu, P.; Jie Liu, J.; Shang, S. The Effects of Electrospinning Structure on the Ion Conductivity of PEO-Based Polymer Solid-State Electrolytes. Energies 2023, 16, 5819. https://doi.org/10.3390/en16155819.
49. Ko, H.J.; Cho, M.H.; Kim, H.-K.; Scanlon, D.O.; Park, H.J.; Choi, Y.-S. Li-ion transport kinetics of Li10GeP2S12 solid electrolyte and its response to isovalent cation substitutions: Density functional theory and machine-learning-assisted molecular dynamics study. J. Alloys Compd. 2025, 1027, 180614.
50. Rom, C.L.; Yox, P.; Cardoza, A.M.; Smaha, R.W.; Phan, M.Q.; Martin, T.R.; Maughan, A.E. Expanding the phase space for halide-based solid electrolytes: Li–Mg–Zr–Cl spinels. Chem. Mater. 2024, 36, 7283–7291. https://doi.org/10.1021/acs.chemmater.4c01160.
